# PD-1/PD-L1 inhibitor-related cystitis: clinical features, diagnostic exclusion, and outcomes in one definite and seven probable cases with an updated literature review

**DOI:** 10.3389/fonc.2026.1905616

**Published:** 2026-08-27

**Authors:** Wei Ding, Heqian Liu, Lin Li, Yingqing Liu, Jiawei Wang, Zhonglang Wang, Lingsong Tao, Xiansheng Zhang

**Affiliations:** 1 Department of Urology, The First Affiliated Hospital of Anhui Medical University, Hefei, Anhui, China; 2 Department of Urology, The Second People’s Hospital, Wuhu Hospital, East China Normal University, Wuhu, Anhui, China

**Keywords:** corticosteroid, cystitis, hematuria, immune checkpoint inhibitor, immune-related adverse event, PD-1, PD-L1, sterile pyuria

## Abstract

**Background:**

PD-1/PD-L1 inhibitor-related cystitis (PD-(L)1-RC) is an uncommon urinary immune-related adverse event that may be mistaken for bacterial cystitis, tumor progression, radiation injury, stone disease, or chemotherapy-related hemorrhagic cystitis. This study describes the clinical phenotype, diagnostic exclusion process, treatment response, and updated literature context of PD-(L)1-RC.

**Methods:**

We retrospectively reviewed eight patients who developed new lower urinary tract symptoms after PD-1/PD-L1 inhibitor-based therapy for malignancies during a prespecified clinical study period from January 1, 2021, to December 31, 2024. Diagnostic evaluation included symptom assessment, urinalysis, urine culture, inflammatory markers, pelvic contrast-enhanced computed tomography or computed tomography urography, and urine cytology, cystoscopy, or biopsy when clinically indicated. Cases were classified as definite or probable PD-(L)1-RC according to the strength of diagnostic evidence. Treatment response was recorded as supportive post-diagnostic evidence rather than a mandatory diagnostic criterion.

**Results:**

The cohort included seven men and one woman, with a median age of 65 years (range, 36-69 years) and a median interval of 5 months (range, 2-36 months) from PD-1/PD-L1 inhibitor initiation to symptom onset. All patients had urinary frequency, urgency, and dysuria without fever. Urine cultures were negative and procalcitonin levels were normal in all patients; seven had leukocyturia, and imaging showed inflammatory bladder changes in all cases. One patient had patient-linked cytological, cystoscopic, and histopathological confirmation and was classified as definite PD-(L)1-RC. The remaining seven patients were classified as probable PD-(L)1-RC. All patients underwent ICI interruption or discontinuation and corticosteroid therapy, followed by symptomatic improvement. Exact CTCAE grade, time to symptom relief, steroid taper duration, ICI rechallenge outcome, and recurrence status were not uniformly documented.

**Conclusions:**

PD-(L)1-RC should be considered in patients receiving PD-1/PD-L1 inhibitors who develop new urinary frequency, urgency, dysuria, sterile pyuria, or hematuria. The seven probable cases in this series were not pathologically confirmed and should be interpreted cautiously. Diagnosis requires structured exclusion of infection, malignancy, radiation injury, stones, urinary tuberculosis, and drug-related cystitis. The proposed diagnostic approach is preliminary and requires multicenter validation.

## Introduction

Immune checkpoint inhibitors (ICIs) targeting programmed death-1 (PD-1), programmed death-ligand 1 (PD-L1), or cytotoxic T-lymphocyte-associated antigen 4 (CTLA-4) have transformed the treatment of many solid tumors. By restoring antitumor T-cell activity, ICIs can also trigger immune-related adverse events (irAEs) involving nearly any organ system (1, 2). Common irAEs affect the skin, gastrointestinal tract, liver, endocrine organs, lungs, and musculoskeletal system; urinary tract irAEs are less frequently recognized.

PD-1/PD-L1 inhibitor-related cystitis (PD-(L)1-RC) is a rare but clinically important urinary irAE. Its typical symptoms include urinary frequency, urgency, dysuria, nocturia, suprapubic pain, and sometimes hematuria. These manifestations overlap with bacterial urinary tract infection, prostatitis, radiation cystitis, bladder tumor, chemotherapy-associated cystitis, stones, urinary tuberculosis, and interstitial cystitis/bladder pain syndrome. Because urinary leukocytes may be present despite negative cultures, patients may receive repeated empirical antibiotics before an immune-mediated etiology is considered. In this manuscript, PD-(L)1-RC refers specifically to cystitis associated with PD-1 or PD-L1 blockade; the broader term ICI-related cystitis/ureteritis is reserved for discussion of the overall checkpoint-inhibitor literature, including rare reports involving CTLA-4 blockade.

This retrospective case series summarizes one definite and seven probable cases of PD-(L)1-RC. The aims were to describe clinical features, diagnostic exclusion, imaging and cystoscopic findings, corticosteroid response, and short-term urinary outcomes, and to place these observations in the context of updated original reports, case series, cohort data, and a recent case-based synthesis.

## Methods

### Study design and patients

This retrospective single-center case series was approved by the Ethics Committee of Wuhu Hospital Affiliated to East China Normal University (approval number: 2022-17-29). Written informed consent was obtained from all participants. The completed clinical dataset covered January 1, 2021, to December 31, 2024. Departmental records were reviewed for patients with documented PD-1/PD-L1 inhibitor exposure followed by new-onset lower urinary tract symptoms suggestive of cystitis. Eligible patients required sufficient clinical documentation to evaluate infection and major structural, malignant, treatment-related, or specific infectious alternatives. Patients whose symptoms were adequately explained by culture-confirmed infection, urinary calculi, tumor invasion or recurrence, pelvic radiation injury, urinary tuberculosis or another specific infection, recent instrumentation, or a clearly implicated bladder-toxic drug were excluded from the definite and probable categories.

### Data collection

Clinical data included age, sex, primary malignancy, PD-1/PD-L1 inhibitor regimen, concomitant anticancer therapy, time from treatment initiation to urinary symptom onset, urinary symptoms, fever, urinalysis, urine culture, blood leukocyte count, procalcitonin level, renal function when available, contrast-enhanced pelvic computed tomography (CT) or CT urography (CTU), urine cytology when indicated, cystoscopic findings, histopathological findings when biopsy was performed, treatment, symptom response, and follow-up urinalysis. Relevant histories of pelvic radiotherapy, urothelial malignancy, urinary stones, and exposure to bladder-toxic chemotherapy were reviewed as part of the differential assessment.

### Diagnostic definition and evidence classification

PD-(L)1-RC was treated as a diagnosis of exclusion. A presentation was considered clinically compatible when it included new lower urinary tract symptoms after PD-1/PD-L1 inhibitor exposure, repeatedly negative urine culture or no clinical evidence of bacterial infection, absent or limited systemic inflammatory response, supportive urinary or imaging evidence of bladder inflammation when available, and adequate exclusion of plausible alternative causes. Evaluation for urinary tuberculosis or another specific infection was guided by epidemiologic risk, imaging, persistent sterile pyuria, and clinical suspicion. Clinical improvement after ICI interruption or immunosuppressive treatment was recorded as supportive post-diagnostic evidence and was not required for initial classification, thereby avoiding incorporation of treatment outcome into the diagnostic definition.

Cases were classified according to diagnostic evidence strength. Definite PD-(L)1-RC required compatible symptoms after PD-1/PD-L1 inhibitor exposure, negative microbiological assessment, exclusion of plausible alternatives, and cystoscopic and/or histopathological evidence of nonmalignant bladder inflammation. Probable PD-(L)1-RC required compatible symptoms, negative urine culture, supportive urinalysis or imaging findings, and exclusion of major alternative causes, but lacked patient-level histopathological confirmation. Cases with insufficient exclusion of infection, malignancy, stones, radiation injury, tuberculosis, or drug-related hemorrhagic cystitis were not classified as definite or probable. Because symptom severity, activities-of-daily-living impact, hospitalization, and intervention requirements were not prospectively recorded in a standardized manner, CTCAE grades were not assigned retrospectively unless the chart contained adequate information.

### Diagnostic evaluation and treatment

All patients underwent clinical assessment for fever, hematuria, dysuria, urinary frequency, urgency, urinary retention, flank pain, and systemic symptoms. The core laboratory evaluation included complete blood count, inflammatory markers, procalcitonin, urinalysis, and urine culture; renal function was reviewed when available. Contrast-enhanced pelvic CT or CTU was used to assess bladder wall inflammation, tumor involvement, calculi, obstruction, ureteral dilation, and hydronephrosis. Urine cytology, cystoscopy, and mucosal biopsy were performed selectively, particularly for gross or persistent hematuria, focal or mass-like bladder wall thickening, previous urothelial carcinoma, persistent symptoms despite initial management, or continuing concern for malignancy. The work-up was tailored to the clinical context rather than applied as an identical invasive protocol to every patient.

The departmental treatment strategy included interruption or discontinuation of the suspected ICI after multidisciplinary consideration and corticosteroid therapy. The observed regimen in this cohort used intravenous methylprednisolone 120 mg/day initially, reduced to 80 mg/day after three days when clinical improvement occurred, followed by conversion to oral dexamethasone and gradual tapering after discharge. This regimen reflected local clinical practice and should not be interpreted as a standardized or universally recommended dose. In future studies and clinical practice, steroid dose and tapering should be individualized according to CTCAE grade, symptom severity, hematuria, ureteral involvement, renal function, infection risk, oncologic benefit, and multidisciplinary assessment.

### Literature review

A targeted literature search was performed in PubMed, Web of Science, and Google Scholar from database inception through July 17, 2026. Search terms combined “immune checkpoint inhibitor,” “PD-1,” “PD-L1,” “CTLA-4,” individual drug names, “cystitis,” “ureteritis,” “cystoureteritis,” “sterile pyuria,” and “hemorrhagic cystitis.” Original case reports, case series, and cohort studies with patient-level or cohort-level clinical information were included; a recent case-based synthesis was retained to define the overall scope of the published evidence. Secondary reviews without new cases, conference abstracts without sufficient clinical detail, duplicate publications, and reports unrelated to urinary tract immune toxicity were excluded. Reference lists of eligible publications were also screened. Because this was an updated targeted narrative review rather than a systematic review, no meta-analysis or PRISMA flow diagram was undertaken, and the reported total should be interpreted as a minimum evidence base rather than an exact registry of unique patients.

### Statistical analysis

Because of the small sample size and descriptive case-series design, no hypothesis testing was performed. Continuous variables are presented as median and range, and categorical variables are presented as number and percentage where appropriate.

## Results

### Baseline characteristics and ICI exposure

The cohort comprised seven men and one woman. The median age was 65 years (range, 36-69 years). Four patients had lung cancer, and the remaining patients had bladder cancer, esophageal cancer, breast cancer, or gastric cancer. PD-1/PD-L1-based regimens included tislelizumab, camrelizumab, atezolizumab, pembrolizumab, and sintilimab, either alone or with chemotherapy or targeted therapy. The median interval from PD-1/PD-L1 inhibitor initiation to urinary symptom onset was 5 months (range, 2-36 months). Patient-level baseline characteristics, diagnostic evidence, and treatment outcomes are summarized in **Table 1**.

**Table 1:** Integrated patient-level baseline characteristics, diagnostic evidence, and treatment outcomes.

No.	Sex/age	Cancer and ICI exposure	Urinary findings	Diagnostic evidence	Treatment and available outcome	Evidence level
1	M, 65	Bladder cancer; tislelizumab; onset 4 mo	Urine WBC >50/HPF; blood WBC 3.12 x10^9/L	CT/CTU inflammation; cytology showed neutrophils without malignant cells; cystoscopy showed inflammatory mucosa without mass; biopsy showed inflammation without malignancy	ICI held/stopped + corticosteroids; symptoms and leukocyturia improved	Definite PD-(L)1-RC
2	M, 52	Esophageal cancer; ABP + nedaplatin + camrelizumab; onset 6 mo	Urine WBC 10-19/HPF; blood WBC 4.35 x10^9/L	CT/CTU inflammation; cytology/cystoscopy/biopsy not documented	ICI held/stopped + corticosteroids; symptoms and leukocyturia improved	Probable PD-(L)1-RC
3	F, 36	Breast cancer; ABP + famitinib + camrelizumab; onset 15 mo	Urine WBC >50/HPF; blood WBC 2.11 x10^9/L	CT/CTU inflammation; cytology/cystoscopy/biopsy not documented	ICI held/stopped + corticosteroids; symptoms and leukocyturia improved	Probable PD-(L)1-RC
4	M, 68	Gastric cancer; camrelizumab; onset 4 mo	Urine WBC 6-9/HPF; blood WBC 2.63 x10^9/L	CT/CTU inflammation; cytology/cystoscopy/biopsy not documented	ICI held/stopped + corticosteroids; symptoms and leukocyturia improved	Probable PD-(L)1-RC
5	M, 58	Small-cell lung cancer; atezolizumab; onset 36 mo	Urine WBC 0-5/HPF; blood WBC 1.53 x10^9/L	CT/CTU inflammation; cytology/cystoscopy/biopsy not documented	ICI held/stopped + corticosteroids; symptoms improved	Probable PD-(L)1-RC
6	M, 65	Lung cancer; carboplatin + ABP + pembrolizumab; onset 2 mo	Urine WBC 30-49/HPF; blood WBC 5.81 x10^9/L	CT/CTU inflammation; cytology/cystoscopy/biopsy not documented	ICI held/stopped + corticosteroids; symptoms and leukocyturia improved	Probable PD-(L)1-RC
7	M, 69	Lung cancer; ABP + nedaplatin + sintilimab; onset 6 mo	Urine WBC 6-9/HPF; blood WBC 7.16 x10^9/L	CT/CTU inflammation; cytology/cystoscopy/biopsy not documented	ICI held/stopped + corticosteroids; symptoms and leukocyturia improved	Probable PD-(L)1-RC
8	M, 67	Lung cancer; ABP + nedaplatin + sintilimab; onset 4 mo	Urine WBC 20-29/HPF; blood WBC 6.50 x10^9/L	CT/CTU inflammation; cytology/cystoscopy/biopsy not documented	ICI held/stopped + corticosteroids; symptoms and leukocyturia improved	Probable PD-(L)1-RC

All patients presented with urinary frequency, urgency, and dysuria without fever; urine culture was negative and PCT was normal in all patients. Treatment response was considered supportive post-diagnostic evidence and was not required for classification. No patient was documented to require cystectomy, long-term bladder drainage, transfusion, or bladder irrigation. No documented ICI rechallenge or recurrence was recorded during available retrospective follow-up. ABP, albumin-bound paclitaxel; CT, computed tomography; CTU, computed tomography urography; HPF, high-power field; ICI, immune checkpoint inhibitor; PD-(L)1-RC, PD-1/PD-L1 inhibitor-related cystitis; PCT, procalcitonin; WBC, white blood cell.

### Clinical presentation, diagnostic evidence, and data completeness

**Figure 1:**
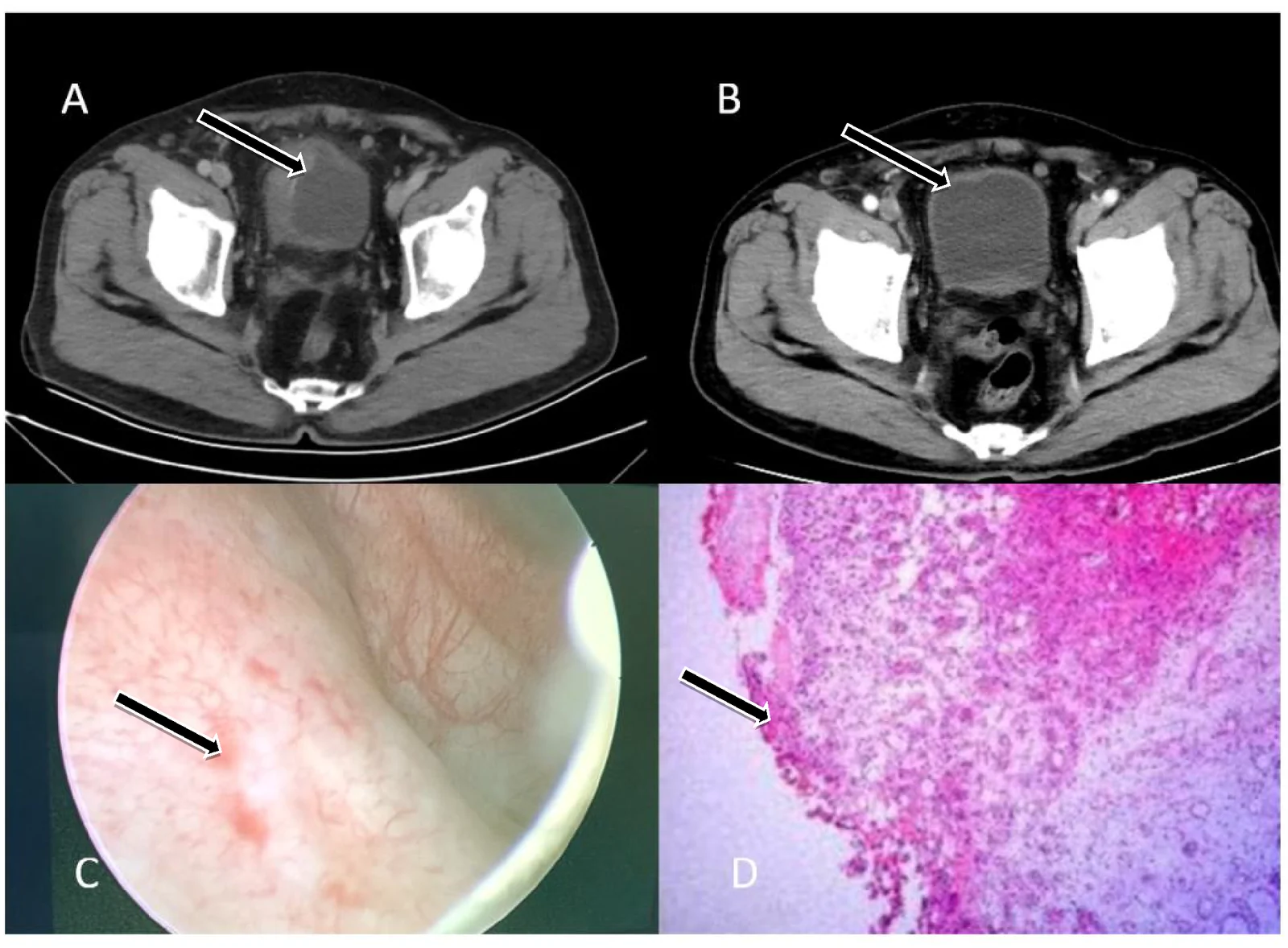
Representative findings from Patient 1 with definite PD-(L)1-RC. **(A)** Pretreatment contrast-enhanced axial CT showing inflammatory bladder wall thickening (high-contrast arrow). **(B)** Follow-up contrast-enhanced axial CT after treatment showing improvement of the inflammatory bladder change (high-contrast arrow). **(C)** Cystoscopic view showing bladder mucosal hyperemia/congestion without a space-occupying lesion (high-contrast arrow). **(D)** Histopathological image showing acute and chronic mucosal inflammation with inflammatory granulation tissue (high-contrast arrow; hematoxylin and eosin staining).

All patients presented with urinary frequency, urgency, and dysuria, and none had fever. Urinary leukocytes were increased in seven patients; the remaining patient had typical symptoms and inflammatory CT findings despite a negative culture. Urine cultures were negative in all cases, blood leukocyte counts were not elevated, and procalcitonin levels were normal. Contrast-enhanced CT showed inflammatory bladder changes in all patients. In Patient 1, the representative case linked to **Figure 1**, urine cytology revealed numerous neutrophils without malignant cells, cystoscopy showed erythematous and edematous mucosa without a space-occupying lesion, and biopsy demonstrated acute and chronic mucosal inflammation with submucosal inflammatory granulation tissue. Patient 1 was therefore classified as definite PD-(L)1-RC. Patients 2-8 were classified as probable PD-(L)1-RC because patient-level pathological confirmation was unavailable; this lower level of certainty is maintained throughout the manuscript.

### Treatment and outcomes

All eight patients underwent ICI interruption or discontinuation after clinical review and received corticosteroid therapy. Urinary frequency, urgency, dysuria, and incomplete emptying improved after corticosteroid initiation. Follow-up urinalysis showed disappearance or marked reduction of leukocyturia among patients with available post-treatment testing. No patient was documented to require cystectomy, long-term bladder drainage, transfusion, or bladder irrigation. Exact CTCAE grade, time to symptom relief, total steroid taper duration, ICI rechallenge outcome, and recurrence monitoring were not consistently recorded. The observed response supported, but did not by itself establish, an immune-mediated noninfectious cystitis phenotype. The retrospective dataset does not permit comparative assessment of steroid dose, taper duration, recurrence risk, or rechallenge safety.

### Differential diagnosis

Because PD-(L)1-RC is a diagnosis of exclusion, alternative etiologies must be actively evaluated, particularly in patients with cancer, previous pelvic therapy, urinary tract malignancy, or hematuria. Key differential diagnoses are summarized in **Table 2**.

**Table 2:** Differential diagnosis of suspected PD-1/PD-L1 inhibitor-related cystitis.

Disease/condition	Clinical clues	Urinalysis/culture	Imaging/cystoscopy/pathology	Differentiating points
Bacterial cystitis or prostatitis	Frequency, urgency, dysuria; fever or pelvic/perineal pain may occur	Pyuria; urine culture often positive; blood WBC/PCT may rise in systemic infection	Bladder wall inflammation; prostatitis may show prostatic tenderness or imaging changes	Responds to appropriate antibiotics; reconsider PD-(L)1-RC when cultures remain negative and antibiotics are ineffective
Bladder tumor or tumor invasion	Hematuria, irritative symptoms, history of urothelial carcinoma or pelvic malignancy	May be normal or show hematuria; culture negative	Space-occupying lesion or focal wall thickening; malignant cells on cytology/biopsy	Cystoscopy and biopsy are decisive when imaging shows focal lesions or persistent hematuria
Radiation cystitis	History of pelvic radiotherapy; delayed hematuria months to years later	Hematuria with or without pyuria; culture negative unless superinfected	Diffuse telangiectasia, friability, fibrosis, or chronic radiation change	Temporal relationship with radiotherapy and cystoscopic pattern help distinguish from PD-(L)1-RC
Chemotherapy-related hemorrhagic cystitis	Exposure to cyclophosphamide, ifosfamide, or other bladder-toxic drugs; hematuria prominent	Hematuria +/- pyuria; culture negative	Diffuse hemorrhagic mucosal injury; pathology nonspecific	Medication history and timing are critical; manage with hydration, mesna when applicable, and supportive care
Urinary tuberculosis or specific infection	Chronic symptoms, sterile pyuria, epidemiologic risk, immunosuppression	Sterile pyuria; acid-fast bacilli/PCR/culture may be positive	Ureteral strictures, contracted bladder, calcifications, granulomatous inflammation	Requires microbiologic testing; corticosteroids alone may worsen infection
Urolithiasis or obstructive uropathy	Renal colic, flank pain, hydronephrosis, microscopic hematuria	Hematuria common; pyuria may occur with inflammation/infection	Stone, obstruction, or hydronephrosis on CT/ultrasound	Relief of obstruction and stone management; ICI ureteritis may mimic obstruction without calculus
Interstitial cystitis/bladder pain syndrome	Chronic bladder pain related to filling, negative cultures, no clear ICI temporal relationship	Usually culture negative; variable pyuria/hematuria	Hunner lesions in a subset; biopsy excludes malignancy	Long-standing pain phenotype and lack of ICI temporal relationship are key clues

### Published evidence summary

The updated literature supports the phenotype observed in this cohort but also underscores the limited evidence base. A 2025 case-based synthesis summarized 43 patients with PD-1/PD-L1 inhibitor-associated cystitis through May 20, 2025 (3). A 2026 report subsequently estimated 46 cases across 29 publications (4), and an additional pathologically supported toripalimab-associated case published on July 14, 2026 (5) increased the minimum published evidence base to at least 47 patients across at least 30 reports by the final search date. These figures should be regarded as minimum counts because of possible overlap among case reports and later syntheses. Recurring patterns included urinary frequency, urgency, dysuria, sterile pyuria or hematuria, negative cultures, inflammatory bladder or ureteral imaging findings, corticosteroid responsiveness, recurrence during steroid tapering or after rechallenge, and occasional ureteritis, hydroureteronephrosis, renal colic, or acute kidney injury (3–28). Most reports involved PD-1 blockade, fewer involved PD-L1 blockade, and CTLA-4 involvement was rare and was described mainly with combined PD-1/CTLA-4 blockade or a bispecific PD-1/CTLA-4 antibody rather than CTLA-4 monotherapy (4, 6, 13). Key reports selected to illustrate these recurring patterns are summarized in **Table 3**; the table is explicitly non-exhaustive.

**Table 3:** Key original and synthesis reports defining the clinical spectrum of checkpoint inhibitor-related cystitis or ureteritis.

Author/year	Study type/no.	ICI exposure	Primary cancer	Main urinary presentation	Management/outcome	Key contribution
Ozaki 2017 (8)	Case report/1	Nivolumab	Pulmonary squamous cell carcinoma	Frequency, dysuria, gross hematuria	Prednisolone; nivolumab later resumed with steroid cover	One of the earliest reports
Shimatani 2018 (9)	Case series/2	Nivolumab	Lung squamous cell carcinoma	Pollakiuria/dysuria, sterile pyuria; recurrence after readministration in one case	ICI discontinuation and/or prednisolone	Supported temporal association and rechallenge recurrence
Ueki 2020 (10)	Case report/1	Pembrolizumab	Lung adenocarcinoma	Pollakiuria, nocturia, dysuria	Prednisolone with clinical and cystoscopic improvement	Pathology showed cytotoxic lymphocytes and increased urothelial PD-L1
Schneider 2021 (13)	Case report/1	Nivolumab + ipilimumab	Metastatic melanoma	Aseptic cystitis	ICI-directed management	Rare report involving combined PD-1/CTLA-4 blockade
Fukunaga 2022 (15)	Case report/1	Nivolumab	Lung cancer	Steroid-dependent or steroid-resistant cystitis	Infliximab achieved control after recurrence	Highlighted refractory disease and second-line immunosuppression
Li 2023 (7)	Case series/3	Tislelizumab, sintilimab, nivolumab	Esophageal and gastric cancers	Urinary irritation, hematuria, hydroureteronephrosis, AKI	Glucocorticoids; urinary and renal improvement	Expanded phenotype to ureteritis and kidney injury
Zhang 2024 (21)	Case reports/2	Sintilimab or pembrolizumab + chemotherapy	Lung squamous cell carcinoma	Hemorrhagic cystitis with frequency, urgency, dysuria, hematuria	Steroids generally required	Emphasized hemorrhagic phenotype
Xiao 2025 (3)	Case-based synthesis/43	PD-1/PD-L1 inhibitors	Multiple malignancies	Broad spectrum of lower urinary tract symptoms	Drug interruption and steroids generally associated with favorable outcome	Defined overall PD-1/PD-L1 case spectrum through May 2025
Yuan 2025 (25)	Case report/1	Nivolumab	Malignancy treated with nivolumab	Nonbacterial cystitis	ICI withdrawal and corticosteroid-based management	Added a recent nivolumab-associated case
Yan 2026 (26)	Case series/3	PD-1/PD-L1 inhibitors	Lung and uterine malignancies	Cystitis/ureteritis with variable upper-tract involvement	Corticosteroid-based treatment	Improved reproducibility through detailed multi-case courses
Wang 2026 (6)	Single-center cohort/12	PD-1/PD-L1 regimens; one PD-1/CTLA-4 case	Multiple solid tumors	Urinary irritation, negative cultures, imaging abnormalities; some AKI	Two spontaneous remissions; ten received steroids; recurrence during taper in some	Largest reported single-center cohort and proposed algorithm
Kong 2026 (28)	Case report/1	Tislelizumab	Small-cell lung cancer	Grade 3 corticosteroid-refractory cystitis	Prednisone + MMF led to resolution	First reported successful MMF use for refractory disease
Peng 2026 (4)	Case report/1	Cadonilimab (PD-1/CTLA-4 bispecific)	Metastatic gastric-type endocervical adenocarcinoma	Imaging-supported cystitis with multiorgan irAEs	Temporary interruption; successful rechallenge without cystitis recurrence	Rare CTLA-4-containing exposure and selected successful rechallenge
Li Z 2026 (5)	Case report/1	Toripalimab + chemotherapy	AFP-producing gastric carcinoma	Gross hematuria, diffuse mucosal hemorrhage, AKI; biopsy with lymphocytic infiltration	High-dose methylprednisolone improved symptoms and renal function	Recent pathologically supported case with AKI

This table is intentionally non-exhaustive and was selected to illustrate early reports, pathologically supported cases, upper urinary tract involvement, steroid-resistant disease, larger case series, and rechallenge outcomes. The updated literature search identified a minimum of 47 published patients across at least 30 reports by July 17, 2026, although exact unique-patient counting is limited by overlap among reports and later syntheses. AKI, acute kidney injury; CTLA-4, cytotoxic T-lymphocyte-associated antigen 4; ICI, immune checkpoint inhibitor; ICI-UC, immune checkpoint inhibitor-related ureteritis/cystitis; MMF, mycophenolate mofetil; TURBT, transurethral resection of bladder tumor.

## Discussion

This case series describes one definite and seven probable cases of PD-(L)1-RC. The shared clinical pattern was new urinary frequency, urgency, and dysuria after PD-1/PD-L1 inhibitor exposure, with negative urine cultures, little systemic inflammatory response, and inflammatory bladder changes on imaging. Sterile leukocyturia was common and may explain repeated empirical antibiotic treatment before an immune-mediated cause is recognized. The principal contribution of the study is a patient-level description of diagnostic exclusion and short-term treatment response, not estimation of incidence, risk factors, or comparative treatment efficacy.

The onset interval in this cohort ranged from 2 to 36 months, indicating that PD-(L)1-RC may occur early or after prolonged treatment. Published reports also extend the phenotype beyond isolated cystitis to ureteritis, hydroureteronephrosis, renal colic, flank pain, and acute kidney injury (5–7, 12, 19, 24, 26). Renal function and upper urinary tract imaging should therefore be considered in patients with persistent symptoms, flank pain, gross hematuria, reduced urine output, or abnormal urinalysis.

Diagnosis remains challenging because no gold-standard test exists and the condition is primarily recognized by exclusion. Cystoscopy and biopsy are especially important when gross hematuria, focal or mass-like bladder wall thickening, previous urothelial carcinoma, persistent symptoms, or poor response raises concern for malignancy. Histopathological findings are generally nonspecific but may support inflammation and, more importantly, help exclude malignancy and selected infections. Reported findings include mucosal erosion, edema, inflammatory granulation tissue, lymphocytic infiltration, plasma cells, eosinophils, and lymphoid follicle formation (5, 10, 14, 23, 27, 29). In the present cohort, only Patient 1 had patient-linked cytological, cystoscopic, and histopathological confirmation. The other seven cases therefore remain probable clinical diagnoses with residual risk of misclassification despite compatible timing, negative cultures, supportive imaging or urinalysis, exclusion of major alternatives, and treatment response.

The updated review also clarifies terminology and scope. Most published urinary irAEs have followed PD-1 blockade, fewer have followed PD-L1 blockade, and CTLA-4 involvement has been reported only rarely, mainly in combined PD-1/CTLA-4 regimens or with a bispecific PD-1/CTLA-4 antibody (4, 6, 13). Accordingly, PD-(L)1-RC is used for the present cohort, whereas the broader ICI-related cystitis/ureteritis terminology is reserved for literature that includes CTLA-4-containing regimens.

Treatment should be individualized according to severity and oncologic context. CTCAE v5.0 classifies noninfective cystitis according to urinary symptoms, hematuria, need for catheterization or bladder irrigation, effects on activities of daily living, hospitalization, transfusion, and life-threatening complications (30). Clinically significant disease commonly prompts multidisciplinary consideration of ICI interruption and corticosteroid therapy (31, 32). The regimen used in this cohort—intravenous methylprednisolone 120 mg/day followed by taper—reflected local practice, achieved symptomatic improvement, and should not be interpreted as a validated standard or universally recommended dose. Recent reports also describe infliximab, JAK inhibition, and MMF in selected steroid-dependent or steroid-refractory cases (6, 15, 28), but these approaches are supported only by limited observational evidence.

Recurrence has been reported during corticosteroid tapering and after ICI rechallenge (3, 6, 9, 11, 18, 23). Conversely, selected successful rechallenges have also been described, including a recent case involving a bispecific PD-1/CTLA-4 antibody (4). Rechallenge decisions should therefore be individualized according to tumor control, available alternatives, severity of the urinary irAE, patient preference, and feasibility of close monitoring. Because rechallenge decisions and recurrence surveillance were not systematically documented in this cohort, the present study cannot estimate rechallenge safety or recurrence risk.

### Proposed clinical workflow

**Figure 2:**
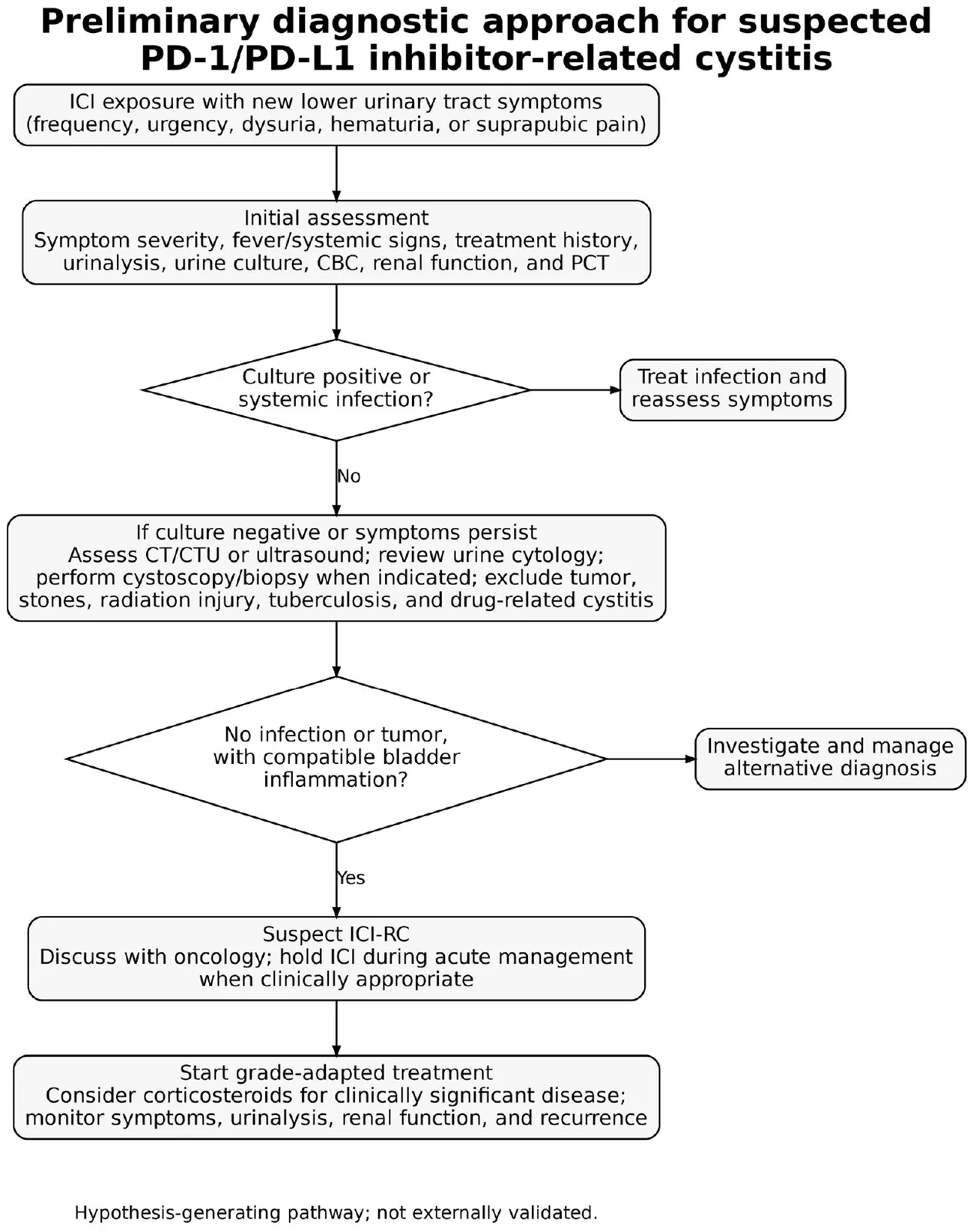
Preliminary diagnostic approach for suspected PD-1/PD-L1 inhibitor-related cystitis. The pathway emphasizes exclusion of infection and tumor involvement, CTCAE-oriented severity assessment when adequate information is available, multidisciplinary discussion before ICI interruption, severity-adapted corticosteroid treatment, and follow-up of urinary symptoms and urinalysis. The pathway is hypothesis-generating and has not been externally validated. PD-(L)1-RC, PD-1/PD-L1 inhibitor-related cystitis; CBC, complete blood count; PCT, procalcitonin; CTU, computed tomography urography.

When a patient receiving PD-1/PD-L1 inhibitor therapy develops new urinary frequency, urgency, dysuria, sterile pyuria, or hematuria, clinicians should assess severity and obtain urinalysis, urine culture, complete blood count, renal function, and inflammatory markers. Culture-confirmed infection should be treated and the response reassessed. If cultures are negative or symptoms persist despite antibiotics, pelvic CT/CTU or ultrasound can assess bladder inflammation, stones, obstruction, hydronephrosis, ureteral disease, and tumor. Urine cytology, cystoscopy, and biopsy should be considered for gross or persistent hematuria, focal or mass-like bladder lesions, previous urothelial carcinoma, persistent symptoms, or concern for malignancy. Probable PD-(L)1-RC may be considered when major alternatives have been adequately excluded and the timing is compatible with treatment exposure. Management should be individualized according to clinical severity, oncologic benefit, patient preference, and multidisciplinary review. This pathway is a practical, hypothesis-generating summary rather than a validated diagnostic or therapeutic guideline. Relevant evidence includes prior reports of immunotherapy-related cystitis and established literature on radiation cystitis, bladder cancer, and urinary tract infection (33–36). The resulting preliminary diagnostic approach is summarized in **Figure 2**.

### Limitations

This study has several limitations. First, it was a retrospective single-center case series with only eight patients, limiting generalizability and preventing formal risk-factor analysis. Second, seven of eight patients lacked patient-linked cystoscopic and histopathological confirmation; despite supportive clinical evidence, misclassification and overestimation of true PD-(L)1-RC therefore remain possible. Third, standardized urinary symptom scores, CTCAE grades, time to symptom relief, steroid taper duration, and long-term recurrence data were not prospectively collected. Fourth, the fixed clinical dataset ended on December 31, 2024, and may not capture later institutional cases, although the literature review was updated through July 17, 2026. Fifth, treatment was not standardized, and the corticosteroid regimen reflected local practice rather than a validated protocol. Sixth, ICI rechallenge decisions and recurrence monitoring were not systematically recorded. Finally, the literature review was targeted rather than systematic; exact unique-patient counting is complicated by overlapping case reports and later syntheses, and the reported total should be interpreted as a minimum.

## Conclusion

PD-(L)1-RC is an uncommon but clinically relevant urinary irAE. This series comprises one definite and seven probable cases and should therefore be interpreted as a description of a compatible clinical phenotype rather than eight pathologically proven events. New urinary frequency, urgency, dysuria, sterile pyuria, or hematuria during PD-1/PD-L1 inhibitor therapy should prompt structured exclusion of infection, malignancy, radiation injury, stones, urinary tuberculosis, and drug-related cystitis. Severity-adapted treatment and careful urinary monitoring may improve outcomes, but larger multicenter studies are required to validate diagnostic criteria, management strategies, recurrence risk, and ICI rechallenge safety.

## Data Availability

The raw data supporting the conclusions of this article will be made available by the authors, without undue reservation.
